# Anti-GQ1b Antibody Syndrome: A Clinician-Oriented Perspective on Diagnostics, Therapy, and Atypical Phenotypes—With an Illustrative 16-Case Institutional Series

**DOI:** 10.3390/jcm15020801

**Published:** 2026-01-19

**Authors:** Taro Bannai, Minako Yamada, Tomonari Seki, Yasushi Shiio, Tatsuya Yamasoba

**Affiliations:** 1 Department of Neurology, Tokyo Teishin Hospital, Tokyo 102-8798, Japan; 2 Department of Otolaryngology, Tokyo Teishin Hospital, Tokyo 102-8798, Japan

**Keywords:** anti-GQ1b antibody syndrome, Miller Fisher syndrome, Bickerstaff brainstem encephalitis, Guillain–Barré syndrome, acute ophthalmoplegia, acute vestibular syndrome, antiganglioside antibodies, molecular mimicry, nodopathy, intravenous immunoglobulin

## Abstract

Anti-GQ1b antibody syndrome (AGABS) unifies triad-defined Miller Fisher syndrome (MFS), Bickerstaff brainstem encephalitis (BBE), and the ophthalmoplegic variant of Guillain–Barré syndrome (GBS-O) under a post-infectious immune mechanism centered on IgG to disialosyl gangliosides. The spectrum also encompasses triad-minus phenotypes—acute ophthalmoparesis without ataxia, acute vestibular syndrome, optic involvement, and acute sensory-ataxic neuropathy. A molecular-mimicry model with complement-mediated nodal/paranodal dysfunction explains severe early deficits despite bland limb nerve conduction studies (NCSs), the cranial/proprioceptive predilection, and generally favorable treatment responsiveness to immunotherapy. In practice, a serology-first strategy, complemented by targeted electrophysiology—blink and H-reflex testing, and, where feasible, paired SEP–ABR showing a literature-supported dissociation (normal ABR with impaired median-nerve cortical SEPs), which, in our series, was documented in one illustrative BBE case—and by structured neuro-otologic examination, mitigates the “normal-NCS trap” and enables timely treatment. Intravenous immunoglobulin (IVIg) is first-line; plasma exchange (PLEX) is an alternative in severe or IVIg-ineligible cases; and intravenous methylprednisolone (IVMP) may be added selectively for central/optic-weighted phenotypes without routine oral taper. We consolidate actionable diagnostic and therapeutic steps and examine them in an institutional series of 16 consecutive seropositive patients (2015–2025): all were anti-GQ1b-positive with frequent GT1a co-reactivity; most reported an antecedent infection—typically upper respiratory, less often gastrointestinal—within the two weeks before onset; limb NCSs were often nondiagnostic whereas reflex/evoked-potential studies were informative; two required intubation in addition to IVIg; outcomes were generally favorable with early immunotherapy. The practical message: order anti-GQ1b at first contact, pair targeted electrophysiology with neuro-otology, and treat early to exploit reversible nodal/paranodal dysfunction.

## 1. Introduction and Scope

Anti-GQ1b antibody syndrome (AGABS) is a clinically actionable construct that unifies triad-based Miller Fisher syndrome (MFS) with Bickerstaff brainstem encephalitis (BBE) and the ophthalmoplegic variant of Guillain–Barré syndrome (GBS-O) under a single immune mechanism centered on immunoglobulin G directed against disialosyl gangliosides [1,2,3,4,5,6,7]. Over the last decade, the umbrella has expanded to include triad-minus phenotypes—acute ophthalmoparesis (AO) without ataxia or areflexia, acute vestibular syndrome (AVS), optic involvement, and acute sensory-ataxic neuropathy—whose recognition depends on pattern-based testing and serology-first strategies [3,5,6,8,9,10,11,12,13,14]. Clinically, individuals can evolve from AO to MFS or from AVS to BBE-like encephalopathy over days without a change in serostatus, underscoring a shared pathophysiologic core [3,5,7,8]. Framing AGABS in this way replaces a rare-eponym mindset with pattern recognition that prioritizes early serology and time-sensitive therapy across emergency, general medical, otorhinolaryngologic, ophthalmologic, neurosurgical, and neurologic settings [1,2,3,4,5,6,14]. We note that the diagnostic recommendations distilled in this article are primarily literature-based and intended to support clinicians in general and emergency settings; our single-center series is provided as an illustrative complement rather than a validation cohort.

## 2. Historical Evolution

The clinical triad of ophthalmoplegia, ataxia, and areflexia was delineated in mid-20th-century descriptions and soon recognized as a coherent syndrome [15]. The paradigm shifted in the early 1990s when serum anti-GQ1b antibody was shown to associate tightly with ophthalmoplegia and ataxia, anchoring the syndrome in molecular immunology [16,17,18]. Through the 2000s, large datasets demonstrated a continuum between MFS and BBE, challenging rigid nosologic walls [7]. In the 2010s, diagnostic classification matured and special phenotypes were captured with greater fidelity—AO without ataxia, AVS with central ocular-motor signatures, optic-predominant disease, and sensory-ataxic forms [6,8,9]. Reports during the pandemic era reinforced a post-infectious framework and accelerated translation of the mechanistic model—molecular mimicry and complement-mediated nodal dysfunction—into day-to-day clinical decisions [1,2,6].

After the identification of anti-GQ1b antibody, diagnostic thinking progressively decoupled from the rigid triad. Large cohorts demonstrated that brainstem encephalopathy with ophthalmoplegia shares serologic and outcome features with MFS, prompting the notion of a single spectrum unified by an antibody rather than separated by clinic-topographic labels [7,18]. Contemporary registry data and narrative syntheses further broadened the spectrum to include AO, AVS, optic-predominant, and sensory-ataxic forms and emphasized that these entities can evolve into one another over days, supporting a dynamic rather than categorical view [3,7,14]. This historical arc explains why a serology-first approach—unusual for peripheral nerve disorders a generation ago—now feels obligatory in AGABS [1,2,3,4,5,6].

## 3. Epidemiology and Triggers

AGABS is reported worldwide with a modest midlife peak and minimal sex skew; apparent demographic differences often reflect phenotype mix and co-reactive antibodies rather than intrinsic biology [1,2,3,4,5,6,7,8,18,19]. Antecedent infections are typical. Upper respiratory tract infections (URTIs) predominate, while gastrointestinal (GI) illnesses contribute a substantial minority; the interval to neurologic onset is usually 1–3 weeks [1,2,20]. Seasonal clustering—winter through spring—likely mirrors pathogen circulation rather than intrinsic disease cyclicity [3,4,20]. A practical pitfall is over-reliance on limb nerve conduction studies (NCSs): early or cranial/afferent-weighted disease can produce severe symptoms despite normal limb NCSs (the “normal-NCS trap”), so clinical pattern and serology should lead [3,4,21].

Incidence estimates remain imprecise because ascertainment depends on the availability of serology and the propensity to test triad-minus phenotypes. Regions with routine testing report higher proportions of AO and AVS within AGABS, suggesting that apparent regional differences partly reflect practice patterns rather than biology [3,4,5,6,8]. Pediatric cases are uncommon but informative: prodromal vomiting and prominent ocular-motor features can precede frank ataxia, and recovery is typically favorable with immunotherapy [7,8,13]. Comorbid autoimmune disease is infrequent and does not appear to change the core clinical–serologic linkage; antecedent vaccination is occasionally reported and should be interpreted within the broader post-infectious framework [2,22]. From a systems standpoint, embedding serology in emergency pathways increases recognition of non-triad presentations and shortens time to therapy [1,2,3,4,5,6,14].

## 4. Pathophysiology: From Molecular Mimicry to Reversible Nodopathy

GQ1b enrichment in cranial nerves III/IV/VI, muscle spindle Ia afferents, and dorsal root ganglia map neatly to ophthalmoplegia, ataxia, and areflexia [16,17,23]. Pathogen lipo-oligosaccharides induce IgG against disialosyl gangliosides, particularly GQ1b, with probabilistic cross-reactivity to GT1a and GD1b [1,6,11,24]. At nodes and paranodes, complement activation disrupts sodium-channel clustering and adhesion complexes, producing conduction failure without overt demyelination—a reversible “nodopathy” [1,25]. This model explains why patients can be profoundly symptomatic when limb NCSs are bland, why cranial/proprioceptive pathways are preferentially affected, and why immunotherapy often produces brisk gains [1,21,25]. GT1a co-reactivity tracks with bulbar involvement and GD1b with sensory-ataxic lean, but treatment should be guided by the whole clinical pattern rather than a single epitope result [6,11,24].

At the microscale, disruption of paranodal adhesion molecules (e.g., contactin-associated protein and neurofascin isoforms) and the sodium-channel cluster reduces safety factors for impulse propagation, yielding conduction failure without demyelinating features on routine studies [1,25]. Complement deposition and membrane-attack complex formation at the node amplify dysfunction but can reverse with timely immunotherapy, matching clinical observations of rapid gains under IVIg or PLEX [25,26,27,28]. Animal and ex vivo models demonstrate that antibody binding can reorganize nodal architecture within hours, supporting the clinical imperative for early treatment when the pattern is recognized [23,25]. The probabilistic mapping between co-reactivity and phenotype (GT1a–bulbar, GD1b–sensory-ataxic) should be interpreted as priors that influence monitoring (e.g., early swallow assessment when GT1a is positive) rather than rigid phenotype labels [6,11,24].

## 5. Clinical Spectrum and Pitfalls

The umbrella incorporates classic MFS/BBE/GBS-O and triad-minus phenotypes: AO without ataxia or areflexia, AVS, optic involvement, acute sensory-ataxic neuropathy, GT1a-aligned bulbar-predominant presentations, and rare extremes including locked-in-like states or vomiting-first pediatric cases [3,5,6,7,8,9,10,11,12,13]. AVS is particularly prone to misclassification because limb strength is often preserved and limb NCSs can be normal early. Central ocular-motor signatures—direction-changing gaze-evoked nystagmus, vertical components, perverted head-shaking nystagmus, downbeat positional nystagmus—and pupillary or eyelid signs should trigger serology and targeted physiology [8]. Explicit attention to visual fatigue, oscillopsia, and motion sensitivity is needed in rehabilitation, as these domains can delay return to normal activity even when limb power recovers [8,19].

## 6. Diagnostics: Pattern-Based Testing in Practice

### 6.1. Principles and Serology-First

Suspect anti-GQ1b antibody syndrome (AGABS) when acute diplopia/oscillopsia or acute vestibular presentations coexist with areflexia or sensory ataxia. Obtain serum anti-GQ1b at presentation; where feasible, add GT1a and GD1b to refine interpretation. In the first week, serology frequently outperforms cerebrospinal fluid (CSF) indices and should not be deferred while awaiting lumbar puncture [1,2,3,6,11,24]. Treatment should not wait for complete confirmatory testing when function is threatened (see Section 6.8.) [26,27,28,29,30].

### 6.2. Serology and Co-Reactivity

Although anti-GQ1b IgG is the serologic anchor of AGABS, additional antiganglioside IgG specificities are not uncommon. Positivity can extend to GM1, GD1a, GT1b, GD3, GalNAc-GD1a, or GD1c, and to ganglioside-complex epitopes (e.g., GQ1b/GT1a, GQ1b/GD1b). These patterns are probabilistic priors rather than rigid labels—GT1a co-reactivity tends to parallel bulbar involvement, whereas GD1b aligns with sensory-ataxic features—yet management must remain phenotype-anchored and time-sensitive. Reports should specify isotype (IgG or IgM), titer, assay platform and cutoff, and calcium-dependence; low-titer isolated IgM or borderline IgG near the cutoff warrants caution and, when available, orthogonal confirmation. Beyond GQ1b/GT1a/GD1b, co-reactive IgG to GD3, GT1b, and GalNAc-GD1a have been described across the GBS–MFS–BBE spectrum and are biologically plausible via shared disialylated motifs and ganglioside-complex formation; interpretation should integrate phenotype and timing rather than serve as an exclusionary rule [6,11,24].

### 6.3. Assay Pitfalls: False Positives and Negatives

Low-titer results near the cutoff or isolated single-antibody positivity without a compatible clinical pattern should be interpreted cautiously; repeat testing and co-reactivity panels help separate signal from noise [9,11,24]. Pre-analytical variables (hemolysis, storage) and biologic factors (transient post-infectious antibodies) can influence results. Conversely, true disease with initially negative serology can occur early; repeating serology after several days may resolve discordance when clinical suspicion is high [1,2,3,6]. Common missteps include anchoring on ocular myasthenia with ptosis/diplopia, diagnosing peripheral vestibulopathy despite central ocular-motor signs, over-weighting a normal early CSF or limb NCS, and delaying therapy while confirmatory tests are pending [8,21,26,27,28,29,30].

### 6.4. Targeted Electrophysiology Beyond Limb NCS

In routine general settings, limb NCSs with F-waves are typically obtained first. Where available, targeted cranial/brainstem studies—blink reflex, soleus H-reflex, and paired SEP–ABR—can be high-yield adjuncts, particularly when limb NCSs are bland and clinical suspicion remains high. Implementation varies with local resources, and findings should be contextualized with serology and bedside ocular-motor examination.

#### 6.4.1. F-Wave Late Responses

F-waves complement limb NCSs and are widely available. Prolonged minimal latencies or absent F-waves may indicate proximal involvement; however, normal F-waves do not exclude AGABS and should not delay serology-first treatment decisions. Document minimal latency and persistence in at least one upper- and one lower-limb nerve to contextualize limb involvement [21].

#### 6.4.2. SEP–ABR Dissociation

Where feasible, obtain paired SEP–ABR to refine localization when limb NCSs are bland; this recommendation is primarily literature-based and, in our series, was documented in one illustrative BBE case. In MRI-negative Bickerstaff-like presentations, a pattern of abnormal median-nerve cortical somatosensory evoked potentials (SEPs) with preserved auditory brainstem responses (ABRs) suggests dysfunction in somatosensory pathways rather than auditory brainstem tracts and is consistent with immune nodopathy [29,30].

#### 6.4.3. Blink and H-Reflex

When standard limb NCSs are non-revealing, blink reflex (pontine circuits) and soleus H-reflex (Ia afferents) may uncover selective cranial/afferent involvement. These abnormalities are reported in triad-positive and triad-minus phenotypes and can precede distal CMAP/SNAP changes in some patients [21,29,30].

### 6.5. Neuro-Otology

In suspected anti-GQ1b antibody syndrome presenting as acute vestibular syndrome (AVS), systematically document ocular-motor signatures at the bedside and, when available, with video-oculography. Direction-changing gaze-evoked nystagmus, vertical components, perverted head-shaking nystagmus, and impaired fixation suppression raise the pretest probability and should prompt anti-GQ1b antibody testing and early treatment consideration when function is threatened [8].

### 6.6. CSF and Imaging; IgG Index and Oligoclonal Bands

Albuminocytologic dissociation—elevated CSF protein with ≤5 leukocytes/µL—supports diagnosis but is timing-dependent and may be absent early; pleocytosis should prompt alternative etiologies [2,4,31]. MRI is primarily for exclusion; a normal MRI does not argue against AGABS, even in BBE-like presentations [5]. Intrathecal IgG synthesis is typically absent; IgG index values are usually within reference range and CSF-restricted oligoclonal bands are uncommon. Measuring the IgG index can help exclude alternative inflammatory CNS disorders in BBE-like cases, but a normal result should not delay treatment when clinical pattern and serology support AGABS [2,5].

### 6.7. Differential Diagnosis and Anchoring

Brainstem stroke, inflammatory encephalitis, myasthenia gravis, Wernicke encephalopathy, ocular myopathies, and non-GQ1b antiganglioside neuropathies are common mimics. When myasthenia is considered, co-order anti-GQ1b to avoid anchoring on a single test or bedside maneuver; discordant data should broaden, not prematurely narrow, the differential [32].

### 6.8. Compact Diagnostic Algorithm (Clinician-Facing)

1. Suspect AGABS when acute diplopia/oscillopsia or AVS coexists with areflexia or sensory ataxia;
2. Order serology immediately: anti-GQ1b antibody at presentation; when available, add anti-GT1a and anti-GD1b antibodies;
3. If limb NCSs are nondiagnostic, add blink reflex and H-reflex; for MRI-negative BBE-like presentations, add median-nerve SEPs and ABRs;
4. Interpret low-titer/early results cautiously; if discordant with a strong clinical pattern, repeat serology and consider co-reactivity panels;
5. Initiate IVIg when function is threatened while confirmatory tests are pending; tailor subsequent testing to refine phenotype and prognosis [8,21,26,27,28,29,30].

## 7. Treatment Strategy: Initiation, Alternatives, and Adjuncts

### 7.1. First-Line Immunotherapy (IVIg)

Intravenous immunoglobulin (IVIg; total 2 g/kg) is the pragmatic first-line across AGABS phenotypes. When functional risk is present—threatened airway, dysphagia, or impending loss of independent ambulation—treatment should not await completion of confirmatory testing [26,27,28]. Standard regimens (0.4 g/kg/day for 5 days, or the same total dose over 2–5 days) are acceptable; delivery should be prompt yet safe. Practical safeguards include dosing by actual/adjusted body weight per policy, slow initial rates with up-titration, screening for renal dysfunction and thrombotic risk, and attention to hydration and venous thromboprophylaxis when indicated. Most patients stabilize or improve within days; a routine second course is not established and should be individualized. If deterioration continues despite adequate dosing—or if IVIg is contraindicated—consider PLEX as an alternative rather than in immediate sequence [26,27,28,33].

### 7.2. Plasma Exchange (PLEX)

PLEX is a valid alternative for severe or rapidly progressive disease or when IVIg is contraindicated [26,27,28,33]. Typical protocols exchange approximately 1–1.5 plasma volumes per session, usually five exchanges over 7–14 days with albumin (±saline) replacement; choice should reflect resources, trajectory, and comorbidities. At the group level, effectiveness is broadly equivalent to IVIg, permitting individualized selection [26,27,28,33]. Sequencing principle: because PLEX removes infused immunoglobulin, avoid initiating PLEX within 24–48 h after IVIg unless there is compelling clinical need; if switching, plan the interval deliberately and document the rationale [26,27,28,33].

### 7.3. Corticosteroids

Intravenous methylprednisolone (IVMP) may be used selectively as an adjunct—for central/optic-weighted phenotypes or severe BBE—when the initial response to IVIg or PLEX is suboptimal. Evidence derives mainly from case reports and small series; no randomized trials support routine use in AGABS, and guidance for classic GBS discourages corticosteroids (oral discouraged; IV weakly discouraged) [2,5,26,27,28,33]. IVMP should therefore not replace immunoglobulin- or exchange-based first-line therapy when function is threatened. If chosen, employ a short, time-limited trial in clinically stable patients with pre-specified objective response checks (e.g., ocular-motor range, dysphagia scales, bedside cerebellar tests) and discontinue within 24–48 h if there is no clear improvement, while proceeding with or continuing IVIg (or considering PLEX when IVIg is contraindicated) [2,5,22,34,35]. In our series (*n* = 3 adjunct IVMP), no serious adverse events occurred. One patient had transient, mild aminotransferase elevation and another had transient, mild hypertension; both resolved without sequelae. No clinically significant glycemic fluctuations were observed, and no infections or thromboembolic events were recorded. If IVMP is used adjunctively, routine monitoring of blood pressure, liver enzymes, and capillary glucose is advisable.

### 7.4. Adverse Effects

IVIg can cause headache, aseptic meningitis, venous or arterial thrombosis, and renal dysfunction; mitigation includes pre- and co-hydration, slower up-titration, and product selection aligned with comorbidity profiles. PLEX requires central venous access and carries risks of bleeding/coagulopathy, hypotension, citrate-related manifestations, electrolyte shifts, and catheter complications; close monitoring and standardized replacement protocols are essential [22,33,34]. Corticosteroids may transiently worsen glycemic control, mood, and blood pressure; their role in AGABS is selective and differs from chronic demyelinating neuropathies [2].

### 7.5. Supportive and Rehabilitative Care

Monitor respiratory mechanics (vital capacity, negative inspiratory force), bulbar function, autonomic stability, and nutrition. Early ocular-motor and vestibular rehabilitation is pivotal to shorten the tail of disability—prism trials for diplopia, gaze-stability exercises for oscillopsia, and graded exposure for motion sensitivity. Avoid vestibular suppressants and sedatives when possible. Provide thromboprophylaxis, multimodal analgesia, and pressure-injury prevention as indicated [22,34].

### 7.6. Relapse and Refractoriness

Relapses are uncommon; repeat IVIg or PLEX is the usual rescue. Persistent or stepwise decline—particularly beyond ~8 weeks from onset—should prompt reassessment for chronic inflammatory demyelinating polyradiculoneuropathy (CIDP) and other causes, guided by time course and electrophysiology [2,36,37].

### 7.7. Emerging Agents

FcRn blockade and complement-pathway inhibition are mechanistically congruent with nodal/paranodal dysfunction. In AGABS, indications, sequences, and endpoints require phenotype-stratified trials before routine use; for now, these agents are promising options in severe or refractory courses within research-oriented or carefully selected clinical contexts [22,25,38].

### 7.8. Airway and Autonomic Management

Even in MFS-dominant presentations, bulbar dysfunction can evolve rapidly. Serial spirometry and bulbar scores, early speech-and-swallow input, and proactive airway planning reduce unplanned intubations. Anticipate autonomic instability—tachyarrhythmias and blood-pressure lability—and titrate therapy cautiously; avoid agents that exacerbate vestibular symptoms when feasible [22,34].

### 7.9. Pain and Rehabilitation

Neuropathic pain is less prominent than in classic GBS but does occur; evidence-based regimens (gabapentinoids, SNRIs) can be used while minimizing sedative burden that worsens oscillopsia. Rehabilitation should include gaze-stability and saccadic training, vergence therapy, graded motion exposure, and pragmatic adaptations (e.g., polarized lenses for photophobia) [8,19].

### 7.10. Disposition and Follow-Up

Early outpatient follow-up (2–4 weeks) to confirm serology, re-measure ocular-motor range, document residual oscillopsia, and titrate rehabilitation increases the likelihood of timely return to baseline activities. Relapse education with clear return precautions is advisable; most relapses, when they occur, respond to repeat IVIg [2,36].

## 8. Prognosis

The overall prognosis is favorable. Many patients improve within weeks to months, and near-complete recovery within a year is common in contemporary cohorts [2,4,31]. Ophthalmoplegia often lags the resolution of areflexia and ataxia, and a subset experiences lingering oscillopsia, photophobia, and motion sensitivity that outlast gains in limb function [3,7,8,19]. Age, initial severity, ventilatory or bulbar compromise, and co-reactivity patterns modulate trajectories [2,4,22,39]. Two practice points merit emphasis: (i) time-to-serology and time-to-IVIg are modifiable determinants of outcome; (ii) explicit management of ocular/vestibular morbidity should be included in discharge and follow-up plans [8,19,22].

Prognostic communication should balance the generally favorable course with candor about the time course of ocular and vestibular recovery. Return to driving or work that demands rapid visual scanning may lag gross motor recovery. Explicit counseling about photophobia and visual fatigue normalizes patient experience and improves adherence to targeted exercises [8,19]. Registries that include patient-reported visual and vestibular metrics will sharpen prognostic estimates beyond global disability scales [22,39].

## 9. Special Phenotypes: Guidance for Otorhinolaryngology (ENT)/Ophthalmology/Emergency

Non-triad presentations lack the comfort of classic patterns. AO without ataxia is often misread as ocular myasthenia; AVS with central ocular-motor signs is easily labeled “peripheral vertigo” [8,32]. Countermeasures are simple and reproducible: maintain a low threshold for anti-GQ1b antibody testing, adopt a neuro-otologic core set at the bedside (direction-changing gaze-evoked nystagmus, perverted head-shaking nystagmus, downbeat positional nystagmus), and deploy blink/H-reflex and SEP–ABR dissociation when MRI is normal [8,21,29,30]. These steps are feasible across emergency, ENT, ophthalmology, and neurology settings and shorten the window during which deficits remain reversible [1,2,3,4,5,6,8,26,27,28].

## 10. Institutional 16-Case Series

### 10.1. Setting and Methods

We retrospectively summarized 16 consecutive seropositive cases managed at our center between 2015 and 2025 (Table 1, Table 2 and Table 3). Extracted elements included demographics; antecedent infection type and interval; phenotype (MFS/GBS/overlap/BBE); initial symptoms and admission features; cerebrospinal fluid (CSF) cell count and protein; immunoglobulin G (IgG) index (IgG index was calculated as (CSF IgG/serum IgG) ÷ (CSF albumin/serum albumin); the laboratory’s upper reference limit was 0.70); neurophysiology (limb nerve conduction studies [NCSs]; blink/H-reflex when available); somatosensory evoked potentials (SEPs)/auditory brainstem responses (ABRs); treatments; and outcomes using the modified Rankin Scale (mRS) at admission and discharge. Free-text reports were conservatively parsed: absence of an explicit “normal” statement was treated as non-classifiable rather than abnormal.

Electrophysiological studies were reviewed against laboratory-specific normative data summarized in Supplementary Table S1. Abnormalities were defined as follows: motor/sensory NCS outside reference ranges; F-wave persistence under the lower limit or inexcitable responses on 20 stimuli; soleus H-reflex absent if non-elicitable at maximal stimulation; median-nerve cortical SEPs and ABR wave latencies/interpeak intervals vs. norms. More than two neurologists independently parsed reports. Neurophysiological studies were performed using a Neuropack system (Nihon Kohden Corporation, Tokyo, Japan).

### 10.2. Cohort Profile, Antecedents, and Timing

Phenotype distribution at admission was MFS 9/16, MFS/GBS overlap 2/16, GBS with ophthalmoplegia (GBS-O) 3/16, and BBE 2/16. Operational rule: we classified GBS-O when limb weakness coexisted with ophthalmoplegia without limb ataxia; cases with limb ataxia were categorized as MFS/GBS overlap. Upper respiratory tract infection (URTI) occurred in 11/16, gastrointestinal (GI) in 4/16, and other in 1/16 cases; one patient had no clear antecedent illness. The interval from antecedent illness to neurologic onset had a median of 14 days (interquartile range 8–14; range 5–16; *n* = 15), consistent with a post-infectious model [1,2,20].

### 10.3. Initial Symptoms and Admission Profile (Re-Audited)

Patient-reported onset was dominated by ocular-motor disturbance (12/16, 75.0%) and distal sensory symptoms (8/16, 50.0%), whereas limb weakness at onset was uncommon (2/16, 12.5%). By admission, the canonical pattern consolidated the following: ophthalmoplegia/diplopia 16/16 (100%), ataxia 11/16 (68.8%), areflexia/hyporeflexia 10/16 (62.5%), sensory symptoms 9/16 (56.3%), pupillary/ptosis findings 8/16 (50.0%), bulbar involvement 5/16 (31.2%), and limb weakness 6/16 (37.5%). These data caution that reliance on limb NCSs or limb weakness alone can be misleading early; a serology-first, pattern-based approach remains pivotal [8,21].

### 10.4. CSF and Neurophysiology

Paired CSF cell count and protein were available in 15/16 cases; albuminocytologic dissociation—defined as CSF protein > 45 mg/dL with CSF leukocytes ≤ 5/µL—occurred in 4/15 (26.7%), consistent with timing effects and the known lag in CSF protein elevation [2,4,31]. The IgG index was available in 14/16 and fell within laboratory reference ranges in all measured cases (median 0.515; IQR 0.49–0.54; range 0.43–0.59); CSF-restricted oligoclonal bands were not reported. Limb NCSs were normal in 15 of 16 patients, and 1 patient showed a motor abnormality (reduced compound muscle action potential [CMAP] amplitude). Reflex physiology was more informative than standard limb studies: F-wave parameters were assessable in 16/16—abnormal in 8/16 and normal in 8/16—and four patients showed absent soleus H-reflexes despite preserved F-waves, a dissociation consistent with selective Ia-afferent/nodal–paranodal dysfunction rather than primary motor axonal failure [21]. In one BBE case with paired testing, ABR was within normative limits while median-nerve cortical SEPs were abnormal; this illustrative finding is consistent with prior reports [29]. Paired SEP–ABR was otherwise not systematically obtained in this retrospective series.

### 10.5. Serology, Treatment Course, and Outcomes

All patients were anti-GQ1b antibody-positive. When available, GT1a co-reactivity was present in 8/16 (50%) and GD1b in 1/16 (6.3%), aligning with reports linking GT1a to bulbar features and GD1b to sensory-ataxic lean [6,11,24]. IVIg was administered in 13/16 (81%). IVMP was used in 3/16 (19%)—all as an adjunct to IVIg—for central/optic-weighted presentations, with no routine transition to oral corticosteroids. PLEX was not used. Spontaneous improvement occurred in 3/16 (19%). Two patients required endotracheal intubation in addition to IVIg (one MFS/GBS overlap, one GBS-O) [26,27,28,33]. At admission, mRS ranked BBE > GBS > MFS; discharge medians improved to 2.0/2.0/1.0, respectively. Exploratory analyses did not detect associations between antecedent type (URTI vs. GI) or interval length and admission/discharge mRS, reflecting limited power rather than evidence of equivalence. Length of stay (LOS) data were available for all 16 patients; the cohort median LOS was 31 days (range 8–93). By phenotype, median (range) LOS was MFS 16 (8–62), MFS/GBS overlap 44.5 (20–69), GBS-O 39 (29–93), and BBE 38.5 (22–55) days. Two intubated cases had longer stays (median 81 days) than non-intubated cases (median 25.5 days). Two patients (2/16, 12.5%) were discharged to an inpatient rehabilitation facility (IRF)—Case 14 (GBS-O) and Case 15 (BBE). Ocular-motor/vestibular morbidity weighed heavily on quality of life, reinforcing early targeted rehabilitation [8,19,22]. Among three adjunct-IVMP cases, no serious adverse events were recorded; one showed transient, mild aminotransferase elevation and another transient, mild hypertension, without clinically significant glycemic fluctuations, and with no infections or thromboembolic events. If IVMP is used adjunctively, routine monitoring of blood pressure, liver enzymes, and capillary blood glucose is advisable (see Section 7.3).

### 10.6. Operational Lessons and Interpretation (Including Phenotype-Specific Physiology)

Two admissions required intubation in addition to IVIg—one MFS/GBS overlap, one GBS-O—and both recovered to ambulatory status by discharge; both exhibited F-wave abnormalities. Three patients improved without immunotherapy, but this should not be extrapolated to a watch-and-wait approach when function is threatened. Given the reversibility of nodal dysfunction, early treatment preserves options and may avoid escalation [1,25,26,27,28]. F-wave abnormalities clustered in BBE (2/2) and were observed in MFS (4/9 evaluable), whereas F-waves were normal in 2/3 GBS-O cases, with the remaining case abnormal; given the small sample, this pattern should be interpreted cautiously—a pattern consistent with cranial/Ia-afferent predilection in MFS/BBE and limb emphasis in classic GBS [21,29,30]. The predominance of normal F-waves in the GBS subset likely reflects a GBS-O pattern with relatively mild early limb involvement—potentially accentuated by the small sample size—whereas the cranial/Ia-afferent predilection in MFS/BBE yielded greater abnormalities on F-waves and the soleus H-reflex—and, where obtained, abnormal median-nerve cortical SEPs with preserved ABRs. IgG index medians by phenotype were within reference ranges (MFS 0.530 [*n* = 9], GBS-O 0.495 [*n* = 2], BBE 0.525 [*n* = 2]); among MFS/GBS overlap, 1/2 had an available index (0.52) and 1/2 lacked a value. Overall, documentation focused exclusively on limb NCS risks obscuring diagnosis in cranial/afferent-weighted disease. Where feasible, inclusion of blink and H-reflex testing, paired SEP–ABR, F-wave parameters, and a neuro-otologic core set would convert ambiguous cases into classifiable ones, support earlier treatment, and improve the quality of datasets used to guide practice [8,21,29,30].

## 11. Practice Implications Across Specialties

Quality improvement metrics could include the proportion of suspected AGABS evaluated with anti-GQ1b antibody within 24 h, the time from arrival to first dose of IVIg when function is threatened, and the percentage of AVS presentations with documented central ocular-motor signs. Tracking these process measures, alongside outcomes such as discharge mRS and time to resolution of ophthalmoplegia, provides actionable feedback loops for teams implementing the pathway.

**Emergency medicine/hospital medicine.** Treat acute diplopia, oscillopsia, and gait unsteadiness as potential autoimmune neuropathy in the right context. Order anti-GQ1b antibody early; when function is threatened, initiate IVIg without waiting for all confirmatory results [26,27,28].

**Otorhinolaryngology (ENT).** In AVS, direction-changing gaze-evoked nystagmus, perverted head-shaking nystagmus, downbeat positional nystagmus, and impaired fixation suppression should trigger anti-GQ1b antibody testing and neurology consultation [8].

**Ophthalmology.** In AO without ataxia, consider AGABS even when neuromuscular junction tests are equivocal, particularly if pupils or lids are involved or if visual fatigue and photophobia dominate [9,32].

**Neurology.** When MRI is normal and limb NCSs are bland, consider targeted blink/H-reflex testing and paired SEP–ABR, where feasible, to refine localization; integrate results with serology and bedside ocular-motor findings [8,21,29,30].

Across all settings, the organizing principle is the same: do not wait for the triad when a recognizable AGABS pattern is present [1,2,3,4,5,6,8,26,27,28].

**Implementation pearls.** (i) Pre-specify anti-GQ1b antibody panels in emergency department (ED) order sets for acute diplopia or AVS with areflexia or sensory ataxia; (ii) train triage nurses and otolaryngology (ENT) and ophthalmology residents to recognize central ocular-motor signatures; (iii) equip ED observation units with bedside tools (Frenzel goggles or video-oculography) to document nystagmus patterns; (iv) and establish rapid-access neurophysiology slots for blink and H-reflex within 24–48 h [1,2,3,4,5,6,8,21].

## 12. Future Research Agenda

### 12.1. Assay Harmonization

Standardize anti-GQ1b antibody measurement and reporting with predefined co-reactivity panels (at minimum GT1a and GD1b) and explicit documentation of isotype, titer, assay method, cutoffs, and calcium dependence; extend harmonization to non-GQ1b targets (e.g., GM1, GD1a, GT1b, GD3, GD1c) and ganglioside-complex epitopes to enable phenotype-anchored meta-analyses and decision thresholds across laboratories. Multicenter ring trials and external quality assessment are desirable to improve interlaboratory comparability [6,11,24].

### 12.2. Neuro-Otologic/Neuro-Ophthalmologic Phenotyping and Early Physiological Markers

Prospectively capture direction-changing gaze-evoked nystagmus, perverted head-shaking nystagmus, and downbeat positional nystagmus—ideally with video-oculography—as a neuro-otologic and neuro-ophthalmologic core set to achieve reproducible phenotyping in AVS and AO and to reduce misclassification of triad-minus presentations [8,21]. Validate the diagnostic and monitoring performance of SEP–ABR dissociation, blink and H-reflex abnormalities, and F-wave parameters across phenotypes, reporting sensitivity, specificity, and treatment dynamics with standardized acquisition protocols [21,29,30].

### 12.3. Treatment Optimization

Conduct phenotype-stratified comparisons of intravenous immunoglobulin (IVIg) versus plasma exchange (PLEX), and define the role of intravenous methylprednisolone (IVMP) in central/optic-weighted disease. Evaluate FcRn blockade and complement-pathway inhibitors in severe or refractory courses with endpoints that include ocular/vestibular function in addition to global disability measures [22,25,26,27,28,33].

### 12.4. Prognostic Modeling, Patient-Reported Outcomes, and Health-Systems Delivery

Develop prognostic models that integrate age, baseline severity, co-reactivity patterns, and targeted physiology (blink/H-reflex, SEP/ABR, F-waves), rather than relying solely on limb NCS or CSF protein, to guide counseling and trial enrichment [2,4,6,22,36,39]. Standardize measures for oscillopsia, visual fatigue, motion sensitivity, and vestibular disability, and embed them in clinics and trials, as these domains map real-world recovery more faithfully than limb strength alone [8,19]. Implement serology-first order sets, reflex testing for GT1a/GD1b, and access to neuro-otology/video-oculography in emergency and subspecialty pathways; train clinicians to recognize the “normal-NCS trap” and to escalate early when function is threatened [1,2,3,4,5,6,8].

## 13. Limitations and Strengths of the Present Synthesis

This Perspective prioritizes clinically actionable guidance and is supported by a single-center series; it is not a meta-analysis. The retrospective design with non-standardized testing—particularly the limited and non-systematic acquisition of cranial-focused electrophysiology (blink reflex, H-reflex) and paired SEP–ABR—may have limited the precision of limb NCS categorization and constrained the granularity of evoked-potential analyses. Our recommendations are primarily literature-based and are intended as pragmatic guidance; the single-center series is presented as an illustrative complement rather than a confirmatory cohort. The cohort is modest, and the lack of detectable associations between antecedent type or interval and outcomes should be regarded as exploratory rather than definitive. External generalizability may be constrained by setting and case mix. Conversely, strengths include a cross-specialty vantage that links mechanistic insight to bedside practice, explicit attention to atypical phenotypes with integrated neuro-otology and neuro-ophthalmology, and a pragmatic algorithm intended to shorten time-to-serology and time-to-IVIg, aligned with contemporary guidance and physiologic data [1,2,3,4,5,6,8,21,26,27,28,29,30]. Finally, while paired SEP–ABR can be diagnostically informative, in this retrospective series it was documented in one BBE case as an illustrative finding; accordingly, our endorsement is primarily literature-based and awaits evaluation under a prospective standardized protocol (under consideration).

## 14. Conclusions

Progress in AGABS will hinge on collaboration beyond neuromuscular clinics. ENT and ophthalmology services are often the first touchpoints; embedding anti-GQ1b antibody testing and standardized neuro-otologic and neuro-ophthalmologic documentation in these settings will improve detection of non-triad phenotypes. On the research side, a federated registry that harmonizes serologic methods, co-reactivity panels, and a concise ocular/vestibular core set would enable adequately powered, phenotype-anchored analyses and pragmatic trials. Such a platform should also capture patient-reported visual and vestibular outcomes that most directly reflect day-to-day function and readiness to return to work.

AGABS is a mechanistically unified yet phenotypically diverse spectrum. Asking early, “Could this be anti-GQ1b antibody?” provides diagnostic leverage disproportionate to the simplicity of the question. A serology-first strategy paired with targeted electrophysiology (blink and H-reflexes, SEP–ABR dissociation, and F-waves) and systematic neuro-otologic examination mitigates the “normal-NCS trap,” shortens time-to-serology and time-to-IVIg, and improves trajectories even when limb strength is preserved. IVIg remains first-line; PLEX is an alternative in severe or IVIg-ineligible settings; IVMP may be considered selectively without routine oral tapers. Our 16-case series aligns with contemporary cohorts and highlights documentation gaps that structured testing can address. Next steps include assay harmonization, neuro-otologic and neuro-ophthalmologic core measures, and phenotype-stratified trials to sharpen diagnosis and personalize therapy [1,2,3,4,5,6,8,9,10,11,12,13,14,21,24,26,27,28,29,30,35].

## Figures and Tables

**Table 1:** Baseline characteristics, clinical diagnosis, antiganglioside serology, antecedent infection, and initial symptoms (*n* = 16).

No	Sex	Age	Clinical Diagnosis	Positive IgG Anti-Ganglioside Antibody	Antecedent Infection Symptoms	Interval (Days)	Initial Symptoms
1	F	34	MFS	GQ1b, GT1a	URTI	14	diplopia the previous day
2	M	35	MFS	GQ1b, GT1a, GD1b	URTI	7	dizziness, unsteadiness, paresthesia, nausea, voiding difficulty from the day of admission
3	M	40	MFS	GQ1b, GT1a	URTI	14	diplopia and paresthesia in both upper limbs for 4 days; unsteadiness from the previous day
4	M	40	MFS	GQ1b	URTI	5	paresthesia in the right side of the face for 2 days; diplopia from the day of admission
5	M	42	MFS	GQ1b (only GQ1b and GM1 were tested)	URTI	16	diplopia and paresthesia in both hands for 8 days; unsteadiness for 3 days
6	M	43	MFS	GQ1b	URTI	7	diplopia and unsteadiness from the previous day; limb paresthesia from the day of admission
7	M	50	MFS	GQ1b	URTI	10	right ptosis for 7 days; diplopia for 2 days
8	F	59	MFS	GQ1b, GT1a	URTI	14	diplopia and right ptosis for 4 days
9	F	72	MFS	GQ1b (only GQ1b and GM1 were tested)	GI illness	10	diplopia for 5 days; bilateral ptosis and unsteadiness from the day of admission
10	M	46	MFS/GBS overlap	GQ1b (only GQ1b and GM1 were tested)	GI illness	14	diplopia for 4 days; headache, nausea, dizziness, and dyspnea for 3 days
11	M	38	MFS/GBS overlap	GQ1b, GT1a	GI illness	14	weakness in both lower limbs for 3 days; diplopia from the previous day
12	F	30	GBS-O	GQ1b	GI illness	10	diplopia and headache for 2 days
13	F	45	GBS-O	GQ1b, GT1a	URTI	14	weakness and paresthesia in both lower limbs for 2 days
14	M	68	GBS-O	GQ1b, GT1a, GD1a, GD3, GT1b	URTI	7	nausea for 2 days; limb weakness from the previous day
15	M	45	BBE	GQ1b, GT1a, GD1a	no	not identified	dizziness for 2 days, followed by dysarthria, paresthesia, diplopia, and limb weakness from the previous day
16	F	64	BBE	GQ1b, GalNAc-GD1a	URTI	14	unsteadiness for 4 days; lower limb weakness and paresthesia in both upper limbs the previous day

Notes: GBS-O = Guillain–Barré syndrome with ophthalmoplegia; URTI = upper respiratory tract infection; GI = gastrointestinal.

**Table 2:** Clinical features on admission and cerebrospinal fluid indices including IgG index (*n* = 16).

No	Clinical Features on Admission	CSF Cell Count (/μL)	CSF Protein (mg/dL)	IgG Index
1	sluggish pupillary light reflex, bilateral ophthalmoplegia	1	23	0.49
2	vertigo, mydriasis, sluggish pupillary light reflex, diplopia, right ptosis, hyporeflexia in all limbs, ataxia	2	62	0.54
3	left ocular movement limitation, bilateral upper limb paresthesia, reduced vibration sense in all limbs, hyporeflexia of all limbs, truncal ataxia	0	26	0.59
4	limitation of left eye abduction, diplopia, oral paresthesia	1	21	0.54
5	bilateral ophthalmoplegia, hyporeflexia in all limbs, paresthesia in both fingers, hyperalgesia, truncal ataxia, mild weakness of the right iliopsoas muscle	1	48	0.47
6	right ptosis, bilateral ophthalmoplegia, bilateral upper limb paresthesia, areflexia of all limbs, truncal ataxia	2	29	0.56
7	mild right ptosis, bilateral ophthalmoplegia, hyporeflexia in all limbs, ataxia	0	79	0.53
8	bilateral ophthalmoplegia, right ptosis, truncal ataxia	1	42	0.51
9	diplopia, bilateral ptosis	1	37	0.43
10	diplopia, mydriasis, unsteadiness, ophthalmoplegia, dysarthria, areflexia in all limbs, limb ataxia	not examined owing to warfarin anticoagulation		
11	diplopia, bilateral lower limb sensory disturbance, limb weakness, paresthesia, hypoesthesia, areflexia, limb ataxia	1	34	0.52
12	headache, diplopia, bilateral ophthalmoplegia, vertigo, dysarthria, dysphagia, dysphonia, limb weakness, truncal ataxia, limb paresthesia	6	38	0.49
13	right ptosis, diplopia, sluggish pupillary light reflex, unsteadiness, proximal limb weakness, bilateral lower limb paresthesia, hyporeflexia	1	35	0.5
14	ophthalmoplegia, dysphagia, limb weakness, areflexia	2	37	not examined
15	mild impairment of consciousness, bilateral abduction limitation, gaze-evoked nystagmus, dysarthria, distal-predominant sensory disturbance in the limbs, limb ataxia, hyperhidrosis	3	39	0.57
16	impairment of consciousness, headache, diplopia, dysarthria, dysgeusia, hyporeflexia, limb weakness, impaired vibration sense in the limbs, paresthesia, ataxia	1	60	0.48

**Table 3:** Neurophysiology (NCS, SEP, ABR), treatment, and outcomes (mRS on admission/at discharge; Length of Stay) (*n* = 16).

No	NCS	SEP	ABR	Treatment	mRS on Admission	mRS at Discharge	Length of Stay (Days)
1	absence of F-wave	normal	not examined	IVIg	2	1	45
2	reduced F-wave responses, absence of H-reflex	not examined	not examined	IVIg	3	1	15
3	normal	not examined	not examined	IVIg	2	1	15
4	reduced F-wave responses	not examined	not examined	spontaneous recovery	2	1	8
5	absence of H-reflex; normal F-waves	slightly prolonged N20 latency	not examined	spontaneous recovery	3	1	16
6	normal	normal	not examined	IVIg	2	1	33
7	normal	not examined	not examined	IVIg	3	2	62
8	reduced F-wave responses	normal	not examined	spontaneous recovery	3	2	15
9	absence of H-reflex; normal F-waves	normal	not examined	IVIg	2	2	56
10	reduced F-wave responses, absence of H-reflex	not examined	not examined	IVIg; intubation and ventilatory support	4	2	69
11	normal	not examined	not examined	IVIg	3	2	20
12	normal	normal	not examined	IVIg + IVMP	3	1	29
13	normal	not examined	not examined	IVIg	3	2	39
14	reduced CMAP amplitudes, reduced F-wave responses	no response	not examined	IVIg; intubation and ventilatory support	5	4	93 (IRF)
15	reduced F-wave responses	N20 not clearly evoked	normal	IVIg + IVMP	5	3	22 (IRF)
16	absence of F-wave	not examined	not examined	IVIg + IVMP	5	1	55

Notes: NCSs = nerve conduction studies; SEP = somatosensory evoked potentials; ABR = auditory brainstem responses (see Supplementary Table S1 for upper/lower limit of normal thresholds and operational definitions); IVIg = intravenous immunoglobulin; IVMP = intravenous methylprednisolone; mRS = modified Rankin Scale; IRF = inpatient rehabilitation facility. Lumbar puncture was not performed in Case 10 owing to warfarin anticoagulation. Cases 14 and 15 were discharged to an IRF.

## Data Availability

The data that support the findings of this study are available from the corresponding author, upon reasonable request.

## Supplementary Materials

The following supporting information can be downloaded at https://www.mdpi.com/article/10.3390/jcm15020801/s1: Supplementary Table S1. Electrophysiology methods and adjudication—operational criteria and laboratory reference limits.

## Abbreviations

The following abbreviations are used in this manuscript:

AGABS	anti-GQ1b antibody syndrome
MFS	Miller Fisher syndrome
BBE	Bickerstaff brainstem encephalitis
GBS-O	the ophthalmoplegic variant of Guillain–Barré syndrome
IVIg	intravenous immunoglobulin
IVMP	intravenous methylprednisolone
URTI	upper respiratory tract infection
GI	gastrointestinal
NCS	nerve conduction studies
SEP	somatosensory evoked potentials
ABRs	auditory brainstem responses
mRS	modified Rankin Scale
LOS	length of stay
